# Gender Differences in Psychological Stress Factors of Physical Therapy Degree Students in the COVID-19 Pandemic: A Cross-Sectional Study

**DOI:** 10.3390/ijerph19020810

**Published:** 2022-01-12

**Authors:** Alberto Bermejo-Franco, Juan Luis Sánchez-Sánchez, María Isabel Gaviña-Barroso, Beatriz Atienza-Carbonell, Vicent Balanzá-Martínez, Vicente Javier Clemente-Suárez

**Affiliations:** 1 Faculty of Sports Sciences, Universidad Europea de Madrid, Villaviciosa de Odón, 28670 Madrid, Spain; alberto.bermejo@universidadeuropea.es (A.B.-F.); isabel.gavina@universidadeuropea.es (M.I.G.-B.); vicentejavier.clemente@universidadeuropea.es (V.J.C.-S.); 2 Department of Medicine, University of Valencia, 46010 Valencia, Spain; beatrizatica@gmail.com; 3 Teaching Unit of Psychiatry and Psychological Medicine, Department of Medicine, University of Valencia, 46010 Valencia, Spain; Vicente.Balanza@uv.es; 4 Centro de Investigación Biomédica en Red de Salud Mental (CIBERSAM), 28007 Madrid, Spain; 5 Grupo de Investigación en Cultura, Educación y Sociedad, Universidad de la Costa, Barranquilla 080002, Colombia

**Keywords:** psychological impact, COVID-19 pandemic, physical therapy students, Spanish

## Abstract

(1) Background: The aim of the study was to investigate how the COVID-19 pandemic impacted the mental health and quality of life of male and female physical therapy students at the European University of Madrid. (2) Methods: A cross-sectional online survey was conducted including a range of tests capturing different domains: 36-item Short Form Health Survey, six-item state version of the State–Trait Anxiety Inventory, Acceptance and Action Questionnaire, Three Items Loneliness Scale, four-item version of the Perceived Stress Scale, Beck Depression Inventory revised version, and Sleep Quality Numeric Rating Scale. (3) Results: A total of 151 students completed the study, consisting of 78 females and 73 males. Gender differences were observed on most of the domains evaluated. Female participants showed worse levels of general health perception, quality of life, depression symptoms, anxiety, stress, experiential avoidance and psychological inflexibility, sleep quality and loneliness compared to male physical therapy students. (4) Conclusions: The results of this study support the need of psychological interventions as preventive programs in situations such as COVID-19 pandemic. The aims of this study comprise of improving knowledge, awareness, and self-coping strategies or other psychological domains oriented to mitigate the effects of COVID-19 on mental health and health-related quality of life in university students, especially among female ones.

## 1. Introduction

As a consequence of the COVID-19 global pandemic, lockdowns were imposed in Spain from March to May 2020 intending to minimize interpersonal contact and avoid the spread of the virus. The virus mainly affects the respiratory system in such an aggressive way that it may require hospitalization or intensive care. This also resulted in a drastic reduction in the mobility and activities of Spain’s population [1,2,3].

In order to reduce the risk of contagions, most governments mandated the use of obligatory facemasks by the general public. However, the use of surgical and N95 masks for a chronic time is related to increased sympathetic modulation due to the increased hypoxia state, that consequently carries increased stress, headaches, impaired motor and cognitive function [4]. Lockdowns may lead to boredom and reductions in reinforcement, which have been associated with physical and psychological symptoms, such as decreased mobility, pain perception, behavioural disorders, sleep disturbances, feeling of loneliness, addictions, depression and even suicides [2,5,6,7]. 

In this way, home confinement imposed by the sanitary situation has produced reduction of the free mobility of people of different ages. This has generated negative consequences at a social, professional and academic level [1,8]. For example, most universities were forced to develop preventive programs such as frequent and regular testing, and the restructuring of the facilities, students and local communities in order to achieve control of the virus transmission [9]. 

Furthermore, academic adaptations including online learning methodologies, remote evaluation along with mask use during lectures could contribute to the loss of affective bonds between students, as well as decreases in their level of concentration and attention during classes [10]. Recent studies have also shown that the use of face masks for 150 min during lectures can affect the mental fatigue perception and reaction time due to an increased heart rate and a decrease in blood oxygen saturation [10]. Altogether, this may lead to an increase in doubts and uncertainty, risk of developing fear, anxiety, acute stress, pain and adjustment disorders in students [1,7,8,9,11].

According to described disorders, gender is an important social determinant of health and gender-based analysis is necessary to improve women’s and men´s health and health care. Worldwide, mental health reveals consistent differences between females and males, with the former displaying internalizing disorders such as depression and psychological distress, while men have, more frequently than women, externalizing disorders, with higher antisocial and substance use disorders [12]. 

In developing countries, these consequences have been more frequently described in women due to different aspects of their emotional attachment and concern for their family well-being, along with a decline in social interaction, which in turn could lead to different mental health complications as outlined [13,14]. According to other population studies, women have poorer mental health status and are more likely to use mental health services than men [15]. Moreover, important gender inequalities have been described regarding behavioural lifestyles and determinants of health [16]. 

Nevertheless, studies assessing the gender differences on the impact of COVID-19 on different domains are necessary in order to expand knowledge about inherent differences and promote gender equity in health [12,13,15,16,17,18]. Knowing the current situation during the COVID-19 pandemic would allow universities and health systems to implement different strategies aimed to improve the mental health and promote healthy lifestyles among university students. For this reason, the purpose of the present study is to analyze gender differences on mental health status in a wide range of psychological stress factors such as anxiety, depression or loneliness among physical therapy degree students during the COVID-19 pandemic. The initial hypothesis was that there would be gender differences, with women presenting significantly higher levels of anxiety and depression than men.

## 2. Materials and Methods

### 2.1. Study Design

This is a cross-sectional, descriptive study in a sample of Spanish physical therapy university students, conducted in accordance with Strengthening the Reporting of Observational Studies in Epidemiology (STROBE) guidelines [19]. 

### 2.2. Setting and Participants

A non-probabilistic convenience sampling was used in this study. An online Google Forms (Menlo Park, CA, USA) survey was mailed to all physical therapy students registered at the European University of Madrid (EUM) by using internal registries. Inclusion criteria for participation were (a) being ≥18 years old and (b) to be enrolled in physical therapy studies in the EUM during the academic year 2020/2021. Data were gathered from 8 to 21 May 2021. From the initial target sample of 1021 students, 151 participants accepted to participate in the study and were therefore included in present analysis. All participants were informed about the objectives of the study and signed online informed consent forms prior to participation. No economic or academic compensation was provided for participation of the students. Participation was kept voluntary and anonymous. Fraudulent activity in the online survey was checked since only completed questionnaires were included in the study. 

### 2.3. Demographic Features of the Sample

Age, gender, academic year, average grade of the student record, nationality and previous COVID-19 infection were self-reported by the participants. 

### 2.4. Endpoints

#### 2.4.1. Health-Related Quality of Life (HRQoL)

HRQoL was assessed through the 36-item Short Form Health Survey (SF-36) [20]. SF-36 consists of 8 scales describing vitality (VT), physical functioning (PF), bodily pain (BP), general health perceptions (GH), physical role functioning (RP), emotional role functioning (RE), social role functioning (SF), and mental health (MH) as well as physical component summary (PCS) and mental component summary (MCS) measures, measured on a scale from 0 to 100. The scales are positively scored, meaning that higher scores indicate better health. Published studies on psychometric properties of the Spanish version of the SF-36 provide sufficient evidence on its reliability, validity and sensitivity [20].

#### 2.4.2. Psychological Measures

Anxiety symptoms were evaluated through the 6-item state version of the State–Trait Anxiety Inventory (STAI-6). The STAI-6 is a 6-item shortened version of the 20-item STAI, but has been shown to be highly correlated to the original scale and good sensitivity to detect group differences in levels of anxiety [21]. The STAI-6 has been used both as a self-administered and online questionnaires [22,23]. The participants rated the frequency of experiencing six emotional states, namely being calm, tense, upset, relaxed, content, and worried, in the last week. A 4-point scale was used (1 = not at all, 2 = somewhat, 3 = moderately, 4 = very much). The scores on the three positively-worded items were reverse coded. Therefore, scores ranged from 4 to 24 (higher values showing worse anxiety status). 

Psychological flexibility was evaluated through the validated Spanish version of the Acceptance and Action Questionnaire II version (AAQ-II) [24]. It is a seven-item Likert scale (total scores ranging from 7 to 49) used to measure experiential avoidance with a good psychometric consistency [24]. The items reflect an unwillingness to experience unwanted emotions and thoughts (e.g., “I am afraid of my feelings,” “I worry about not being able to control my worries and feelings”), the inability to be in the present moment and behave towards values-directed actions when experiencing psychological events that could undermine them (e.g., “my painful experiences and memories make it difficult for me to live a life that I would value,” “my painful memories prevent me from having a fulfilling life,” “worries get in the way of my success”). 

Stress was reported using the 4-item version of the Perceived Stress Scale (PSS-4), which uses general questions about psychological stress rather than focusing on a specific experience [25]. PSS-4 has been identified as a useful tool when conducting online screening [26,27]. The sensitivity of the PSS-4 is a positive aspect of the scale and has been normed internationally [28]. Participants were asked to report on their thoughts and feelings of control over life events and confidence in dealing with these experiences during the last month. Responses are given on a Likert-type scale from 0 (never) to 4 (very often). PSS-4 scores are obtained by summing together the scores of the four questions, with the middle two items being reverse scored. 

Depression symptoms were assessed by using the Beck Depression Inventory revised version (BDI-II) [29]. It is a self-report scale with 21 items and a score ranging from 0 to 3; the total score is obtained by summing each item. It has been validated in Spanish university students with a good internal consistency and adequate psychometric properties [29]. According to the application manual, BDI-II score levels are minimum (0–11), mild (12–19), moderate (20–35), and severe (36–63). 

#### 2.4.3. Lifestyle Outcomes

Loneliness was assessed through the Three Items Loneliness Scale (TILS) [30], that captures self-perception of social connectedness. It is a reduced version from the UCLA Loneliness Scale by indexing the items with the highest loadings in the loneliness factor. Participants were asked how often they felt: (1) lacking companionship; (2) were left out; and (3) were isolated from others, on a 3-point Likert scale coded from 1 ‘hardly ever’, to 3 ‘often’. Individuals’ responses were summed up, with higher scores indicating greater loneliness. The validated Spanish version has shown adequate sensitivity and was used in this study [31].

Sleep quality in students was assessed through the single-item Sleep Quality Numeric Rating Scale (SQ-NRS) [32]. Participants were asked to report the quality of their sleep over the past 24 h on an 11-point numeric rating scale ranging from 0 (“best possible sleep”) to 10 (“worst possible sleep”). This scale has demonstrated positive psychometric properties in different populations [32]. 

#### 2.4.4. Statistical Analyses

Means (±standard deviation or interquartile range) and absolute frequencies (%) were used to describe demographic characteristics of the sample. Normality of the variables was checked through the Shapiro–Wilk test. Afterwards, sex differences in both demographic characteristics and psychological-health related outcomes were analyzed by the T-student test or the U-Mann–Witney test and the Chi-square test when it applied. All analyses were performed using SPSS Statistics for Windows, Version 22 software (IBM Corp., Armonk, NY, USA). Statistical significance was set at an alpha value of 0.05. 

## 3. Results

We included 151 participants between 18 and 54 years old with a mean age of 23.39 ± 5.18. Male were 51.66% while 48.34% were women. We did not find gender differences in demographic characteristics in terms of age (male = 23.51 ± 5.13 vs. female = 23.27 ± 5.27), nationality distribution, share with previous COVID-19 infection (25.64% vs. 26.03%). We did not find differences in the proportion of male/female subjects in the physical therapy studies overall (44.74% vs. 55.26%, *p* > 0.05). Results are described in Table 1.

According to HRQoL and psychological health-related outcomes, we found significant sex differences in the scores of the domains of the HRQoL in SF-36 test evaluating physical functioning (male = 96.2 ± 6.8 vs. female = 93.5 ± 9.0; *p* = 0.036), vitality (male = 62.8 ± 20.3 vs. female = 48.6 ± 20.6; *p* < 0.001), social function (male = 74.7 ± 25.3 vs. female = 61.9 ± 22.8; *p* = 0.003), emotional (male = 60.7 ± 42.9 vs. female = 41.1 ± 42.5; *p* = 0.006) and mental health (male = 69.7 ± 18.6 vs. female = 56.1 ± 21.2; *p* < 0.001), with female participants showing significantly lower HRQoL levels.

In addition female participants showed worse scores in the STAI-6 (male = 12.4 ± 4.3 vs. female = 15.5 ± 4.5; *p* < 0.001), the AAQII (male = 19.1 ± 8.3 vs. female = 25.2 ± 10.3; *p* < 0.001), TILS (male = 4.1 ± 1.3 vs. female = 5.1 ± 1.3; *p* < 0.001), PSS-4 (male = 5.2 ± 3.2 vs. female = 7.5 ± 3.5; *p* < 0.001) and BDI-II (male = 15.2 ± 10.6 vs. female = 25.6 ± 12.4; *p* < 0.001) scores, compared to male participants. 

## 4. Discussion

The present study aimed to examine the gender differences on mental health status in psychological stress factors such as anxiety, depression symptoms or loneliness among physical therapy degree students during the COVID-19 pandemic. Our study showed the existence of gender differences on the domains of overall health, vitality, social function, emotional and mental health with female participants showing lower HRQoL levels than male participants. Furthermore, female participants showed lower levels of sleep quality, and worse scores in anxiety symptoms, perceived stress, experimental avoidance, loneliness, and depressive symptoms compared to male participants in our sample.

To our knowledge, this is the first study to provide a comprehensive description of some of the main psychological risk factors involved in mental health and quality of life among physical therapy university students. Previous research found a correlation between stress and quality of life of university students to maintain and/or improve their personal satisfaction and academic performance [33]. However, analyzing gender differences in stress perception between participants represents one of the novelties of the present study, but did not analyze gender differences in perception between participants. 

According to the literature, female gender has been identified as a risk factor for higher psychological impact during stressful situations such as the COVID-19 pandemic [14]. In our study, higher perceived stress was observed among female physical therapy students. Although these gender differences could be related with the effect of the pandemic, they converge with those previously described in the general population samples [34], university students [35], and, more specifically, health science students [36]. Subsequent studies should clarify the relationship between the perceived stress and the COVID-19 pandemic in this population. Prior work in this area shows that pandemic situation impacted significantly on the student experience of academic life, exacerbating social inequalities and impacting on quality of life. Previous qualitative studies [9] have explored students perceptions and experiences of social distancing and self-isolation. Regarding quality of life, health sciences students have been shown to report worse mental health and total HRQoL scores than other university students [37]. In our study, male students consistently scored higher than females in most domains of HRQoL, including vitality, social functioning, emotions and mental or general health. Although differences in general have been found in previous studies [38], gender differences in the rest of the domains of the perceived HRQoL represents a novelty of the present study.

In this study, females reported more depressive and anxiety symptomatology, and higher psychological inflexibility, and higher perception of loneliness or social isolation and psychological stress. These results converge with those of previous studies that analyzed mental health among university students and, more precisely, health sciences students [39,40]. Previous surveys suggest the potential risk for a less healthy lifestyle in those undergraduate students with a history of mental illness in the previous year or a positive screening for depression and anxiety [18,41]. Therefore, there may be a relationship between the perceived low quality of life (e.g., vitality) and poor mental health reported by female students in our sample. Psychological inflexibility and experiential avoidance are theorized to contribute to the development, maintenance and exacerbation of a broad range of psychological problems such as mood and anxiety disorders, substance use disorders, eating disorders, and psychotic disorders [42,43]. However, further research is needed to explore the role of the different factors contributing to mental health among health sciences students during the COVID-19 pandemic.

Our results are in line with other studies that highlight the importance of avoiding the rigid dominance of psychological reactions over chosen values and contingencies in guiding action in life, in other words, being psychologically flexible. Higher psychological flexibility is positively associated with an increase in well-being and quality of life [44], whereas avoidance behaviours are directly proportional to psychological disorders [2].

These results are important because they suggest that female physical therapy university students present a higher prevalence of mental and physical health problems. Of those, most are stress-related problems, which are caused by accumulation of damage. Therefore, even if this population is young, it is expected that women will develop stress-related health problems over time [34]. 

Several studies have found that poor mental health is associated with lifestyle in undergraduate students [45,46]. It is worth noticing that the effect size of the differences between men and women in previously described psychological endpoints are medium-large (close to Cohen’s d = 0.8), supporting the importance of these findings. Not surprisingly, women reported worse quality of sleep than men already. Our results are similar to those by Du et al. [47] who found a positive correlation between perceived stress and sleep quality, and a negative correlation between stress and resilience in a multi-country sample of students during COVID-19 pandemic. In this line, sleep quality and psychological resilience are considered two factors that have the potential to alter the relationships between stress and health behaviors [47]. During the COVID-19 pandemic, online learning substituted the face-to-face learning environment on campus. It is known that online learning environments are challenging for some students and could contribute to loss of social support, and enhance social isolation and stress perception in this population [9].

The present study results showed greater levels of loneliness perception among women compared to men that could contribute to worse outcomes in stress, anxiety, or depression endpoints. These findings are consistent with previous studies that underline the central role of loneliness and social connections in health [48,49]. To our knowledge, no previous study has analyzed gender differences in loneliness among health sciences students.

In sum, those findings are also consistent with the perception of control in health. Indeed, perception of lack of control to obtain the desired quality or quantity of social connections, or keep aligned with one´s values, leads to psychological stress and therefore, the risk of multiple stress-related problems [50], including anxiety, depression, and, as result, impaired quality of life [33,45]. 

The current study adds to the literature suggesting the importance of implementing and developing psychological services and intervention programs for university students across all countries with a high impact of COVID-19 pandemic [51,52]. Different levels of external support could help university students, especially women, to better cope with stressful live events, and ultimately improve wellbeing, and decrease stress, anxiety and depression levels [45]. Loneliness in this population could be mitigated through increased intra-university communications and a focus on establishment of low transmission-risk social activities to help students build and enhance their social support networks [9]. Previous studies suggest that technology-based interventions as apps are an appropriate and effective delivery modality to facilitate social connection, increasing mental health and reducing loneliness [51]. In addition, very brief (e.g., one hour) growth mindset and social belonging interventions can have prolonged positive effects on student well-being and achievement [51].

Other interventions might include preventive programs directly delivered by healthcare workers, telephone-based and online consultations as well as online free self-help manual for students, with the aim of improving knowledge, awareness, and self-coping strategies or other psychological domains. Interventions should be implemented in order to improve physical, psychological and mental health in this population.

However, this study presents with some limitations such as its relatively low participation rate and sample size or lack of comparison with other degrees or universities around the world which may reduce the external validity of the study. Non-response bias and common method bias was not envisioned in our study, and it is considered as a limitation, but we acknowledge that this study was performed by online survey during a pandemic situation by a sample selection for convenience, which precluded us to included further demographic data so as to compare included-non-included participants. Furthermore, the cross-sectional design provides data from a single point in time, which does not allow us to establish the evolution of variables analyzed over time, and its observational nature prevents inferring causal relationships. In addition, the use of qualitative statistical methods in these kinds of studies could have helped to complement and improve the analysed results, although only a quantitative test was used. Lastly, research based on self-reported data could favour information bias due to the social desirability effect.

## 5. Conclusions

In summary, this study, conducted during the global COVID-19 pandemic, showed gender differences in the impact on domains of health such as general health perception, quality of life, depression, anxiety, stress, experiential avoidance and psychological inflexibility, sleep quality and loneliness in a sample of physical therapy university students. These findings highlight the need to implement strategies oriented to psychological health improvement, especially among female university students, in situations of stress such as the COVID-19 pandemic.

## Figures and Tables

**Table 1:** Comparison between male and female participants.

Variable	Gender	N	Average	Standard Deviation	T	*p*	Confidence Interval Lower Upper	
Age (years)	Male	78	23.51	5.15	−0.495	0.621	−0.35457	0.21256
	Female	73	23.27	5.27				
SF-36 physical functioning	Male	78	96.21	6.75	2.10	0.036	0.1697968	5.279799
	Female	73	93.49	9.04				
SF-36 role limitations: physical	Male	78	91.98	21.88	1.99	0.047	0.1043865	18.11655
	Female	73	82.87	33.30				
SF-36 Pain	Male	78	71.43	20.75	0.563	0.574	5.011605	9.00669
	Female	73	69.43	22.82				
SF-36 General health perceptions	Male	78	76.46	15.79	2.879	0.005	2.64596	14.22232
	Female	73	68.02	20.07				
SF-36 energy/vitality	Male	78	62.82	20.36	4.250	0.000	7.59240	20.78836
	Female	73	48.63	20.65				
SF-36 social functioning	Male	78	74.67	25.32	3.060	0.003	4.49589	20.89048
	Female	73	61.98	22.82				
SF-36 role Limitations: emotional	Male	78	60.68	42.89	2.817	0.006	5.84683	33.32891
	Female	73	41.09	42.49				
SF-36 mental health	Male	78	69.69	18.60	4.204	0.000	7.22684	20.04819
	Female	73	56.05	21.24				
SF-36 declared health evolution	Male	78	3.28	0.97	−0.303	0.763	−0.35177	0.25834
	Female	73	3.32	0.91				
SQ-NRS	Male	78	6.41	2.06	1.966	0.050	−0.00374	1.49244
	Female	73	5.67	2.55				
STAI-6	Male	78	12.35	4.34	−4.366	0.000	−4.59246	−1.73069
	Female	73	15.52	4.55				
AAQII	Male	78	19.08	8.29	−4.052	0.000	−9.11856	−3.14031
	Female	73	25.21	10.25				
TILS	Male	77	4.07	1.32	−3.600	0.000	−1.53580	−0.44734
	Female	73	5.06	1.32				
PSS-4	Male	78	5.16	3.19	−4.188	0.000	−3.36362	−1.20715
	Female	73	7.45	3.51				
BDI-II	Male	78	15.16	10.59	−4.493	0.000	−12.12662	−4.71813
	Female	73	23.58	12.41				

Abbreviations: SF-36: 36-item Short Form Health Survey; SQ-NRS: Sleep Quality Numeric Rating Scale; STAI-6: six-item state version of the State Trait Anxiety Inventory; AAQII: Acceptance and Action Questionnaire II version; TILS: Three Items Loneliness Scale; PSS-4: four-item version of the Perceived Stress Scale; BDI-II: Beck Depression Inventory revised version.

## Data Availability

All data is presented in the study.
